# The burden of care, parenting stress, and navigating welfare services: parents’ everyday experiences of young children with autism spectrum disorder

**DOI:** 10.3389/fpsyt.2026.1841274

**Published:** 2026-06-30

**Authors:** Gunn Karin Brechan-Skjetne, Ragnhild Bjørknes, Maj-Britt Rocio Posserud, Rannei Sæther

**Affiliations:** 1 Regional Centre for Child and Youth Mental Health and Child Welfare (RKBU), Department of Mental Health, Faculty of Medicine and Health Sciences, Norwegian University of Science and Technology, Trondheim, Norway; 2 The Habilitation Service for Children and Young People, St Olavs Hospital, Trondheim, Norway; 3 Regional Centre for Child and Youth Mental Health and Child Welfare, Norwegian Research Centre AS (NORCE), Bergen, Norway; 4 The Norwegian Center for Child Behavioral Development (NUBU), Oslo, Norway; 5 Department of Child and Adolescent Psychiatry, Division of Psychiatry, Haukeland University Hospital, Bergen, Norway; 6 Department of Clinical Medicine, University of Bergen, Bergen, Norway; 7 Gilberg Neuropsychiatry Centre, University of Gothenburg, Gothenburg, Sweden; 8 Department of Rehabilitation Science and Health Technology, Faculty of Health Sciences, Oslo 23 Metropolitan University, Oslo, Norway

**Keywords:** autism, autism spectrum disorder, family, parenting stress, parents, preschool, welfare state

## Abstract

**Background:**

Parenting a child with autism spectrum disorder (ASD) is demanding and affects all aspects of life, yet parents’ experiences during the child’s early years remain underexplored, especially from Scandinavian countries. This study examined parents’ experiences in a Scandinavian context characterized by strong parental involvement of both parents, extensive preschool coverage, and comprehensive welfare systems. Our aim was to explore how parents of preschool children experience everyday parenting and how these experiences shape parenting stress and family life.

**Materials and Methods:**

Thirteen individual semi-structured interviews were conducted with mothers and fathers of children with ASD aged three to five. This study is part of the “Enabling Parents of Children with Autism Spectrum Disorders - A Randomized Controlled Study on Parenting Programs”, registered at Clinical-Trials.gov (ID: NCT05750095). Data were analyzed using Systematic Text Condensation, a descriptive and exploratory cross-case thematic approach.

**Results:**

Three main categories were identified: “*Everyday family life”, “Family and social networks”*, and *“Meeting the system in daily life”*. Parents described continuous adaptation to their child’s needs; everyday life required continuous follow-up while managing concerns of siblings and the child’s safety. Experiences of participation and isolation coexisted, and parents frequently fostered understanding and acceptance of ASD while seeking practical and emotional support in everyday life. Preschool services and support were important. In their interactions with welfare services, parents often encounter bureaucratic complexity when seeking competence, stability, and flexibility.

**Conclusion:**

Parenting a young child with ASD is a dynamic process involving ongoing tasks, adaptation, and learning, strongly shaped by both the child’s needs and the coherence of the surrounding support systems. When services are fragmented, insufficient, or uncoordinated, the parental burden and stress increases, whereas moments of mastery and support foster resilience, underscoring the need for competent, flexible, and family−adapted services.

## Introduction

Autism spectrum disorder (ASD) is a neurodevelopmental condition that typically emerges in early childhood (1) and has significant implications for the child, the parents, and overall family functioning (2). Prevalence estimates indicate that approximately 1% of children receive an ASD diagnosis (3), with higher rates reported among boys (4, 5). ASD is characterized by difficulties in communication and social interaction, alongside repetitive behaviors and restricted interests (1). Many children also present with sensory hypersensitivity (1, 6), sleep disturbances (7), and selective or restricted eating (8, 9) and some display challenging behaviors such as intense tantrums, self-injury, or aggression (10, 11). Early−emerging characteristics often prompt parental concerns about developmental delay or atypicality and initiate the diagnostic process (10). Taken together, these early and often persistent characteristics can create a complex everyday caregiving context that places substantial demands on parents.

At the same time, parenting offers positive and enriching experiences, while these often coexist with challenges that contribute to stress. Parenting stress refers to the imbalance between parenting demands and resources, conceptualized as a product of child characteristics, parental factors, and the broader socioeconomic environment (12, 13). Research consistently demonstrates that parents of children with ASD report substantially elevated levels of parenting stress and a diminished quality of life (14–16). These outcomes are frequently associated with a range of complex and interacting challenges, including navigating an often long diagnostic process, managing the child’s aggressive behaviors and episodes of self-injury, coping with sleep disturbances, and meeting extensive, ongoing demands related to daily caregiving (15, 17, 18). Parenting stress is further intensified by limited support, financial strain, and work-life conflicts (15, 17). Parents also face social isolation, perceived stigma in society, and strained family relationships, underscoring the well-established need for social support (15, 19, 20). Such challenges place significant demands on families, affecting daily routines, parental functioning, and sibling well-being (21). Some parents report that typically used practices are insufficient to meet their child’s needs (22). Parent education and support groups are highlighted as key resources that can help reduce stress, restore a sense of balance, and foster empowerment (15, 17, 19, 23–25).

Although several qualitative studies have explored parents’ experiences of raising a child with ASD, most include parents of children across a wide age range, often extending into late childhood or adolescence (up to 12 or even 18 years) (17, 19, 26–28). Consequently, the preschool period has received relatively limited attention. While some reviews have addressed related topics, such as parental stress, family functioning, diagnosis, or stigma, these either focus on specific aspects of parenting or include broad age ranges that extend beyond the preschool years (17, 19, 26–30). Despite these contributions, knowledge about parents’ everyday lived experiences during the preschool period remains limited. To our knowledge, no qualitative synthesis has specifically focused on this group and on everyday life experiences in this early developmental phase.

This gap is important, as the early years are often described as particularly demanding, both practically and emotionally, and may involve a need for targeted information, coordinated services, and trust in professional support (31, 32). However, how these demands are experienced in parents’ daily lives during the preschool period remains underexplored.

Building on this knowledge, it is also essential to situate parental experiences within the broader Scandinavian context. In this region, family life is shaped by dual−earner households and high parental involvement of both parents (33). Within this context, gender equality is a central policy aim, supported through generous parental leave schemes and substantial paternal involvement (34, 35). Scandinavian societies also offer comprehensive public childcare arrangements. Preschools provide early childhood education and care for children aged 1–5/6 years, with a particular emphasis on inclusive, play-based pedagogical approaches that promote learning, development, and psychosocial well-being throughout the day (36). Most young children attend preschool, and many receive special educational support when needed (37, 38). Competent staff such as pedagogical leaders, special education teachers, multidisciplinary teams collaborate with parents, and both preschools and families may access further consultation and guidance from municipal educational services (36). These practices are embedded in the Scandinavian welfare model, which promotes quality through universal access to coordinated municipal and specialist services (39, 40).

In the Norwegian context in particular, high maternal employment, substantial paternal leave policies, and near−universal preschool enrollment are combined with municipality and specialist health services that offer treatment, guidance, and competence development for families (34, 35, 37, 39). Families facing elevated caregiving demands may receive respite services and care coordination to support family functioning (41). Together, these structural features illustrate how Nordic welfare arrangements aim to ensure universal access and robust disability support for families of young children with ASD (42).

To our knowledge, existing Scandinavian qualitative research on parents of preschool children’s experiences primarily focuses on diagnosis and interactions with support services, such as preschools and welfare systems (32, 43–47). Studies from Denmark and Sweden describe parents’ emotional responses to diagnosis, its importance for accessing support, and their efforts to navigate service systems. They also highlight the need for competent preschool staff, individualized support, and coordinated, long-term follow-up across services (32, 43–47).

Despite these insights, limited attention has been given to how parents experience everyday life while raising a young child with ASD. To our knowledge, no Scandinavian or Nordic studies have specifically focused on parents’ everyday experiences in the preschool period. Therefore, the aim of the present study was to explore how Norwegian parents of young children with ASD experience everyday parenting and how these experiences shape parenting stress and family life.

### Theoretical framework

This study draws on developmental and contextual frameworks that illuminate how parenting unfolds across everyday life contexts. Bronfenbrenner`s relational and contextual understanding of child development emphasizes that parenting is shaped across everyday life domains, and children’s development and family experiences are influenced by interactions across multiple environmental levels, from close relationships to broader societal systems (48, 49). The Abidin model of parenting stress (12, 50) highlights the imbalance between demands and resources, situating parenting stress as a product of child characteristics, parental factors, and the broader socioeconomic environment.

## Materials and methods

The present study used a qualitative research design and conducted semi-structured interviews with parents. The study was part of the Norwegian study “Enabling Parents of Children with Autism Spectrum Disorders - A Randomized Controlled Study on Parenting Programs” (ENACT). ENACT is a multicenter study involving four participating clinics and is registered in ClinicalTrials.GovP nr ID: NCT05750095 on January 26, 2022.

### Recruitment and participants

We used a combination of convenience and purposive sampling to recruit parents who had recently consented to participate in the ENACT study but had not yet started the parenting programs. The inclusion criteria for the ENACT study were being the parent of a child with ASD aged two to six years and being sufficiently proficient in the Norwegian language to participate in the parenting program. Clinicians at the sites informed and invited the parents to participate in an interview. Purposive sampling was used to achieve a broad sample in terms of children and parents and to enhance representativeness of the population.

Thirteen parents participated, including eight mothers and five fathers, representing diverse families. The parents’ mean age was 36, ranging from 26 to 47. The informants were recruited from three clinics and resided in urban and rural areas. Some parents were single (3), while others lived with a partner (10). The study included parents from diverse ethnic backgrounds with varying professions and educational levels.

The mean age of the children was 4 years (ranging from 3 to 5 years old). Parents of both boys (11) and girls (2) were represented. Four children did not have siblings, while the others had various positions within their sibling groups. The children were diagnosed between 3 months and 2.5 years before the interviews. They had varying levels of ASD; more than half of the children did not have any vocal verbal language. All children attended preschool.

### The interview guide

The interview guide was developed based on Abidin’s (12, 50) model of parental stress and inspired by theory from Bronfenbrenner (48, 49). A literature review, clinical experiences, and discussions in the research team also influenced the guide. The team consisted of authors GKBS, RB, MBRP, and RS with backgrounds in pediatrics, child psychiatry, psychology, physiotherapy, child welfare, and social education. They had expertise in ASD, family work, and qualitative analysis. The interview guide was structured around the central question: “How do you experience being a parent of a child with ASD?” Supplementary questions examined parenting, family dynamics, and sources of parenting stress by addressing child and parent characteristics and the broader socioeconomic context. Parents were encouraged to reflect on everyday challenges, the impact of autistic symptoms, their parenting role, and factors shaping family well−being. The interview guide was reviewed by a parent associated with a Norwegian ASD advocacy organization and piloted with another parent, resulting in minor refinements. The complete interview guide can be found in the **Supplementary File**.

### Data collection

The interviews were conducted between August 25, 2023, and March 4, 2024, by the first author (GKBS). Most took place at the clinics (n=9), while some were done digitally (n=3) or by phone (n=1). Interviews lasted on average 53 minutes (range: 37–79 minutes). They were arranged to provide a calm setting and allowed for breaks if needed. All the recorded interviews were transcribed verbatim and anonymized.

### Data analysis

Analyses were conducted using the Systematic Text Condensation (STC) procedure, an exploratory and descriptive thematic cross-case analysis of qualitative data (51). The analysis followed an inductive, data-driven approach. No predefined theoretical framework guided the coding or condensation process. Rather, themes were developed from participants’ accounts. Relevant theoretical perspectives were subsequently drawn upon in the discussion to interpret and contextualize the findings. All authors contributed to the analysis.

In the first step, all authors independently reviewed all the transcripts to establish an overview and identify preliminary themes. They then met to discuss and develop preliminary themes through consensus. Differences in interpretation were explored and negotiated through discussion. Consensus by authors was achieved after two meetings, finalizing this first step with five preliminary themes: routines/ predictability, other adaptations in everyday life, the family, social life and activities and support services.

In the second step, GKBS systematically reviewed all transcripts line by line, identifying and sorting codes or meaning units (a term in STC that defines a text fragment containing information) (51) into the five preliminary themes identified in the first step. In the subsequent coding process, the meaning units were identified, classified, and grouped into preliminary themes, which were now developed into codes groups. A codebook was created, and the features and labels of the code groups were adjusted during the coding process. With significant changes, the dataset was reviewed again. Some meaning units or codes were assigned to multiple code labels. The authors discussed codebook and the material collaboratively.

In the third step, the codes in the code groups were sorted into subgroups. Each subgroup was then the unit of analysis. During the following condensation, the codes in the subgroups were reviewed, and the content was summarized into a condensate, fabricated quotation that preserves, to the greatest extent possible, the original terminology used by the participants. Borderlines and content of the code groups and subgroups were adjusted through this process. Quotations that illustrated the content of the condensate were chosen. The material and the choice of quotations were discussed among the authors.

In the fourth and last step, the content of the condensates was synthesized and transformed into results. The material was reorganized into relevant categories, and new topics were developed. The results and quotations were then translated into English. GKBS reviewed all transcripts to validate the results. The analysis team discussed and agreed upon during the final stages of the analysis. Consensus by authors was achieved through joint discussions. Saturation was considered achieved when further analysis yielded redundancy rather than new insights.

The team emphasized reflexivity, critically reflecting on preconceptions, roles, and practices. This included reflection on how preconceptions, roles, and prior experiences could influence the analysis. Preconceptions were written down prior to analysis and actively reflected upon throughout the analytic process. The researchers reflected on their dual roles as professionals and researchers, and how these positions might influence interpretations. The first author’s involvement in conducting all interviews and much of the analytic work was subject to reflexive consideration throughout the analysis. NVivo software was used to analyze and manage the data, and a codebook was developed to facilitate the identification of themes and codes.

### Ethics

The study was approved by the Western Norwegian Regional Ethics Committee (No.18894). Parents provided written informed consent and received a study summary, also available on the project website. All materials were in Norwegian. Participants were informed of the interviewer’s role as a social educator, clinician, and PhD candidate in the ENACT project. Parenting programs began shortly after interviews, with optional follow-up sessions to address any concerns.

## Results

Analyzing the parent interviews led to three categories: “*Everyday family life”, “Family and social networks”*, and *“Meeting the system in daily life”.* The categories reflect progression from the innermost sphere of family life, through relational experiences in extended networks, to interactions with public services and systems. Many of the answers from the parents ran across each category. Each category is comprised of subcategories as described in detail below (see **Table 1**), supported by illustrative quotes from the interviews. The results section presents data-near, descriptive findings, while theoretical interpretation is reserved for discussion.

**Table 1:** Summary of categories and subcategories.

Category	Subcategory
Everyday family life:	Facilitating structure in daily routines and managing stress Worrying about siblings Ensuring safety
Family and social networks:	Experiencing participation and isolation Promoting understanding and acceptance Seeking emotional and practical support
Meeting the system in daily life:	Accessing support and services in preschool Seeking competence, stability, and flexibility in a fragmented system Navigating bureaucracy to access services

### Category 1: Everyday family life

#### Facilitating structure in daily routines and managing stress

Parents emphasized strategies for meeting their child’s ongoing needs and adapting family life. Continuous one-to-one engagement required interpreting subtle cues, anticipating reactions, and adjusting responses, an iterative process that fostered parental expertise. Caregiving was often easier alone, allowing for undivided attention. Predictability and routines were considered essential for regulation and stress reduction, despite the effort required. Tools such as verbal prompts, visual aids, and step-by-step instructions supported transitions, with morning and evening routines described as particularly demanding. One parent described the process: “The same things should happen every morning … [Name] needs more time, especially in the transitions” (Parent 11).

Despite these efforts, some children developed rigid interaction patterns or strong attachments to specific family members, which were easily disrupted by changes such as illness, absence, or the arrival of a sibling. Parents described this as emotionally exhausting, noting that the child’s needs often dominated family life. One parent shared: “It’s all about [Name] … Everything revolves around what the child wants, and it feels like we’re being controlled. [Name]’s home and [Name] are constantly directing us, telling us what to do” (Parent 4). Another parent described how the child regulated who was allowed to be present: “[Name] is happy with me sitting on the sofa … Lately, the child likes to have the big sister in the living room, but not so much the big brother … He’s being chased out of the living room” (Parent 13).

#### Worrying about siblings

Parents expressed concern about the situation’s impact on siblings, particularly regarding time, attention, and emotional availability. Balancing the needs of a child with ASD and siblings was described as a persistent challenge, often requiring divided caregiving roles or compromises during family time. As one parent put it: “[Name] gets 75% of the time, and she gets 25%” (Parent 2). Parents feared siblings might feel neglected or burdened, as they frequently had to wait, step aside, or manage independently, even when needing support themselves. One parent expressed concern like this: “I have to forget the two older ones when I am with [Name]” (Parent 8).

Parents expressed concern about potential long-term effects but hoped siblings would eventually develop understanding. Fostering these relationships required patience and adaptation to the child’s needs. Parents facilitated siblings’ interpretation of behaviors and emotions. For infants, sensory sensitivities necessitated gradual adjustment, while shared interactions typically emerged through familiar, physical play. One parent reflected:

The child has always had a good relationship with us, but not with the siblings. It`s become such a pain in the soul … However, over the last six months, it has begun to loosen up. They interact by jumping and playing together on the trampoline. It has been a long process, constantly explaining to the siblings, “I think [Name] really loves you, right?” The child doesn’t show that, and it has been a bit tough for siblings (Parent 12).

#### Ensuring safety

Parents frequently expressed profound concern for their child’s safety, emphasizing the child’s limited understanding of danger due to ASD-related challenges. Unlike their peers, these children often failed to recognize hazardous situations, necessitating constant supervision and care. One parent explained, “You must be 110% present; you never know what the child is up to” (Parent 12). Parents described an intense, continuous vigilance and that they sometimes knew what the child was doing even from another room. High activity levels and strong motor skills, combined with poor risk awareness, made everyday environments, such as those involving water, traffic, heights, or sharp objects, particularly perilous. Despite repeated efforts to teach safety skills, children often struggled to generalize these skills across various contexts or respond effectively to instructions. A parent described it like this:

The child doesn’t look around; doesn’t look for the cars like we’ve talked about a million times. The child doesn’t stop when we say “stop”. The child doesn’t wait when I say “wait” and can go straight out into the road (Parent 1).

Parents described frightening situations where the child suddenly disappeared from home, garden, or public places without warning, not following instructions, or responding to their name. Some had installed extra locks at home, but safety measures like gates and fences were often outsmarted. Parents lived in constant alert, unable to relax, always fearing the worst. One parent shared:

I lost [Name] once at a mall … I called out, but the child didn’t answer … I ran up to the guard and said, ‘Can you see where [Name] is on the surveillance cameras?… The child just sprinted up an escalator … I try to avoid taking the child alone to the mall and other places (Parent 6).

### Category 2: Family and social networks

#### Experiencing participation and isolation

Children’s challenges influenced family participation in social and daily activities. Some families managed with minor adjustments like providing support, reading signals, or leaving early, while others avoided events such as birthdays or holidays due to them being overwhelming. Organized activities were often too demanding, whereas informal, active outings offered relief. To reduce stress, parents prioritized according to the child’s needs and the event’s significance, opted for short visits, used calming tools, or hosted gatherings at home to maintain control. As one parent put it: “We can’t visit our great-grandmother because everything will be broken. I must go there alone, or she must see them at our place” (Parent 1).

Parents reported that participating in social or community events, involving peers and other parents, was challenging, stressful, and emotionally painful. One notable example was attending preschool-related gatherings: “I was in the hallway with [Name] and the staff, but then I had to take the child outside. I left and decided not to participate next year” (Parent 13). For some, everyday life became so demanding that it led to withdrawal and isolation, especially in the early years. One parent reflected: “We were isolated without being fully aware … We avoided social situations … and we still feel that we are a little outside of society” (Parent 3).

#### Promoting understanding and acceptance

Some parents reported challenges in extended family relationships due to limited knowledge of ASD. Parents often shared information and materials from courses or professionals to help relatives understand the child’s needs. They emphasized that parenting a child with ASD required distinct approaches and a different set of parenting skills. One parent expressed: “I feel there is no point in seeking advice from my mother” (Parent 8). While some family members adapted, adjusting expectations and accepting atypical behaviors, others relied on assumptions or misinterpreted social difficulties. Parents described some family members’ acceptance as gradual and emotionally complex. In some cases, relatives withdrew, believing the child was uninterested, leaving parents to mediate and interpret. One parent shared: “[Name] ‘s aunt kept her distance to avoid crossing limits because [Name] didn`t look at her, and she thought [Name] wasn`t aware of her. I had to explain that the child talked about her after she left” (Parent 8).

Beyond the family, parents also worked to foster understanding in broader social contexts. They frequently explained the child’s behavior to other parents and children, particularly in preschool or in playgounds. One parent shared: “It can be scary for them, especially if they are younger children or if they don’t know [Name], right? (…) How much can you really explain to a three- or four-year-old, that [Name] just wanted to play with you. I find that difficult” (Parent 5). Explaining was described as a necessary but emotionally demanding process. One parent shared own experience from informing other parents early in preschool:

We feel we must inform them so they can understand when [Name] is pulling the pants on parents we don’t know. And they need to know that [Name] doesn`t have any language and might not understand so much as their child … It’s painful, but they appreciate us letting them know (Parent 10).

#### Seeking emotional and practical support

Emotional support from friends, family, and peers was vital for parents’ well-being. Some disclosed the child’s diagnosis early, while others hesitated, fearing invalidation or emotional strain. One parent shared: “With some friends, I was very open from the very beginning. These are the ones it’s easiest to talk to now. So, cry and tell from the beginning, then they will travel the journey with you” (Parent 4). Supportive individuals, especially those with relevant experience, became key allies, offering guidance or a safe space to share concerns. Parents valued connecting with others raising children with ASD through personal networks or organizations some of them also offering activities with the child: “It’s more about there being a place that is adapted to my child—somewhere we can go knowing that it won’t be awkward for anyone, and that people understand if [Name] screams or shouts a bit…” (Parent 9).

Practical support, particularly childcare, was harder to access; relatives often felt unable or unsecure to take care of the child. Parents hesitated to ask and reserved this for urgent situations. One parent recalled a time when no help was available during a crisis: “It’s only in crises that we ask … A close family member died … I had no choice but to bring both children to the funeral” (Parent 1). Detailed instructions were necessary when others stepped in, especially regarding routines and communication. Still, misunderstandings could occur, leaving parents anxious about entrusting their child’s care to others. One parent explained:

I had explained things like: “When the child says…, it means…’ She looked after the child for two days and didn’t understand what [Name] was saying … but they didn’t want to call me because I had time off” (Parent 6).

### Category 3: Meeting the system in daily life

#### Accessing support and services in preschool

Parents reported varied experiences with preschool services. Supportive staff, especially special needs educators, were valued, and ASD-specific competence and early intervention, even pre-diagnosis, were seen as critical. Feeling understood and receiving regular feedback fostered trust and shared purpose. As one parent put it: “To hear what the preschool has done for [Name], how far they have reached, what they are doing, it gives me light at the end of the tunnel” (Parent 13). Preschool offered practical and emotional support when staff demonstrated commitment. As one parent shared:

I know that I can come to the preschool and the public nurse and ask them about the strangest things … Also, they say: “Yes, you are a good mother”. It makes me confident that I’m doing what’s best for the child (Parent 7).

Yet concerns persisted about meeting individual needs. Morning and afternoon transitions were challenging, and some observed greater progress during holidays, questioning educational content and sensory demands. Uncertainty, poor structure, and weak communication led to guilt, mistrust, and hesitation. One parent reflected: “When [Name] is not with us, how do people understand and interpret? And how can [Name] express wants and needs?… For 7½ hours, right?” (Parent 12). Another parent expressed frustration: “Many hours of follow-up in preschool, but does it help? …. I miss a plan and get annoyed. I ask for a plan. … Getting feedback is essential … I no longer trust the system and explore other options” (Parent 2).

#### Seeking competence, stability, and flexibility in a fragmented system

Parents emphasized the importance of competent services to support both child and family. ASD-specific expertise and effective communication were seen as essential, yet many felt their own insights were undervalued. Variations in competence and resources across preschools and municipalities contributed to unequal service quality. One family described how initial judgment and insufficient support were replaced by demonstrated competence, strengthened parental involvement, and practical facilitation of the child’s development:

Your child doesn’t talk; it’s your fault. You don’t read to the child; you don’t do your thing … The first time we visited the new preschool, they gave me pictures on posters: “Please bring these with you; they can help”… In one year, [Name] has gone from 0 to 100. It’s amazing. I did not believe it could happen. [Name] speaks now (Parent 7).

Stability in caregiving and services was a major concern. Parents sought predictable, long-term arrangements and valued professionals with emotional capacity and practical experience. Trust developed slowly, while unmet promises and inconsistent support caused frustration. Staffing changes or preschool routine shifts disrupted the child’s stability, often forcing parents to adjust work schedules and even threatening the child’s health. One parent described the effects of staff changes: “[Name] gradually stopped eating. Didn’t want breakfast in preschool, a bit for lunch, and no meals in the afternoon. Then [Name] stopped drinking” (Parent 10).

Flexibility was crucial; families needed services adapted to routines, work demands, and individual needs. Rigid structures, limited hours, and long travel distances created barriers, prompting calls for personalized solutions tailored to their family’s needs such as in-home care or shorter respite periods. One parent described this frustration:

The help is a choice of either sending the child away on Friday and getting it back on Sunday, or nothing. I think it’s very unreasonable … It’s not the best for [Name]; it has to start in another way. I think it shows a lack of knowledge, and it’s one solution that must fit everyone (Parent 1).

#### Navigating bureaucracy to access services

Parents described navigating the welfare system as exhausting and emotionally draining, often facing frustration, isolation, and repeated applications within a fragmented structure. While some questioned the availability of supportive services: “What welfare state?” (Parent 1), others emphasized the need for proactive coordination and a clear point of contact, such as a child coordinator. One parent shared how overwhelming they felt after receiving the diagnosis:

I miss someone contacting us; a crisis team is very dramatic, but we felt we came home with sort of a death notice and had no one to talk to. At the same time, you have a long list of things to find out. It felt like everything was falling apart. So, it would have been helpful if the child coordinator had been prepared and if there was a system where specialist health services contacted the municipality services and informed them that we had just given a family a serious message (Parent 3).

Securing help required persistence, extensive paperwork, and sometimes appeals to supervisory authorities. Formal diagnoses occasionally eased access, yet unmet promises, inconsistent follow-up, and difficulty finding suitable helpers added to the emotional toll. One parent reflected on the struggle to secure necessary services:

I don’t know if we are the first in that preschool or what it is, but it is a very, very large fight to get the adjustments that the child needs … It’s a 50–60 percent part-time job to apply, follow up, and fight for services. I have checked, and I think we sent 160 pages of paper to the municipality and the Norwegian Labor and Welfare Administration (NAV) (Parent 12).

## Discussion

The study explored how parents of preschool−aged children with ASD experience everyday parenting and how this shapes parenting stress and family life within the Scandinavian context. Everyday family life involved structuring routines, managing stress, worrying about siblings, and ensuring the child’s safety, all of which required sustained attention and adaptation. Turning to family and social networks, parents described experiences of participation and isolation, efforts to promoting understanding and acceptance among relatives and peers and seeking emotional and practical support to manage the demands of daily life. Finally, parents described experiences of navigating welfare systems, including accessing support and services in preschool, seeking competence, stability, and flexibility in a fragmented system, and managing bureaucratic requirements to obtain necessary resources.

This study draws on Bronfenbrenner’s ecological theory conceptualizing parenting and child development shaped through dynamic interactions across multiple environmental systems (48, 49) and Abidin’s model of parenting stress arising from an imbalance between parenting demands and available resources (12, 50).

### The burden of care and parenting across ecological layers

**Figure 1:**
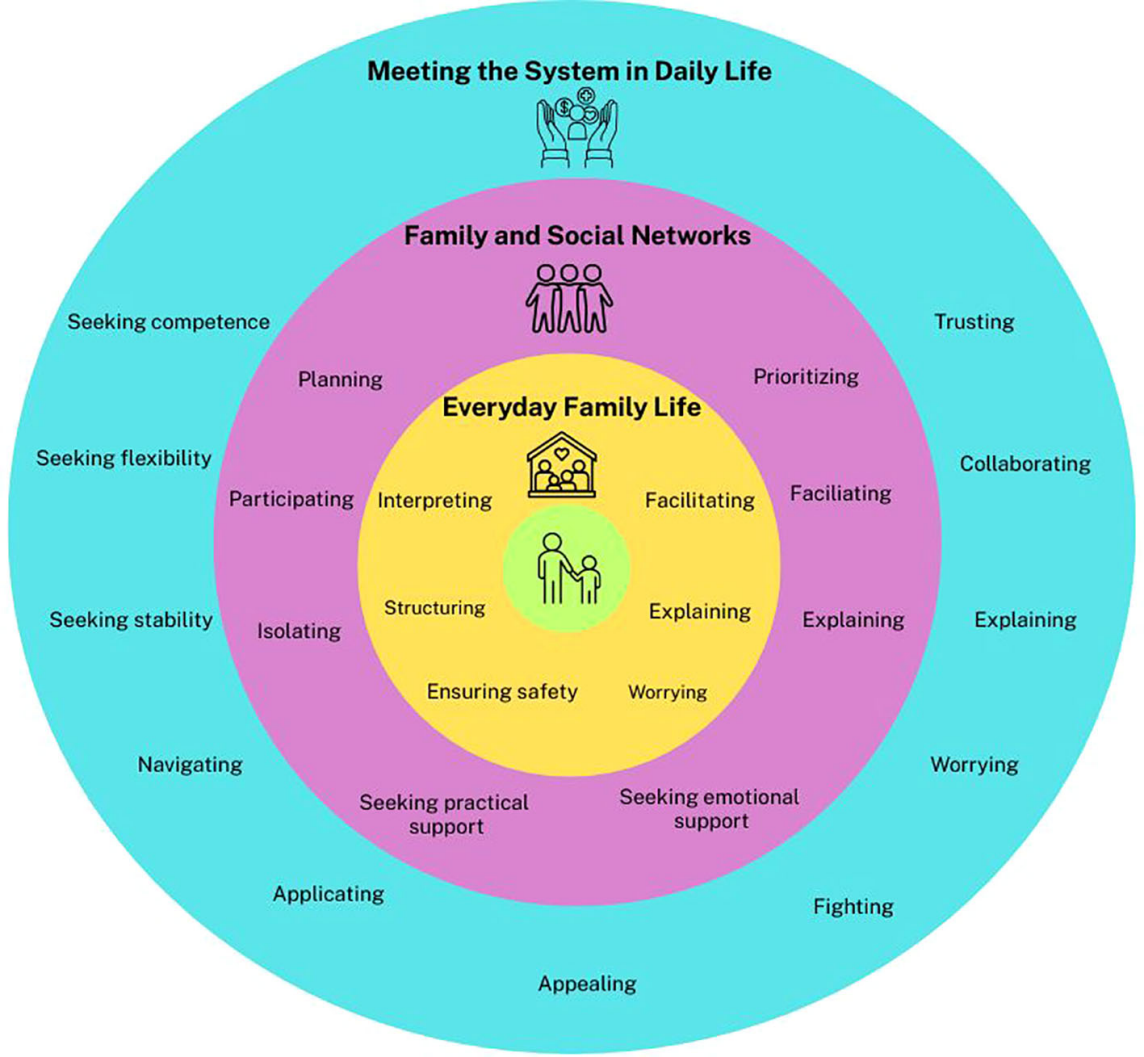
The burden of care. Source: Developed by the authors in https://canva.com, inspired by Bronfenbrenner’s ecological systems theory (Bronfenbrenner, 1979) (48).

The findings illustrate the extensive caregiving responsibilities and cumulative burden of care carried by parents, which can be meaningfully interpreted through Bronfenbrenner’s ecological systems theory (48, 49). At the microsystem level, parents continuously interpret their child’s experiences, structure everyday routines, ensure safety, and manage family- and sibling dynamics. The mesosystem becomes evident in how parents coordinate, negotiate, and sustain connections between home, extended family, social networks, and the child’s preschool. This involves planning and prioritizing social participation, fostering understanding and acceptance of the child’s ASD, and seeking emotional and practical support from others. At the exosystem level, parents interact with preschools and welfare services as institutional actors, engaging in collaboration, advocacy, and occasional appeals as they navigate systems that directly influence their child’s and family’s opportunities. Our analysis sheds light on the substantial overall caregiving burden parents’ shoulder, reflected in the wide range of everyday “doings” they engage in to support their child (See **Figure 1**).

The overall caregiving burden also relates to parenting stress as described by Abidin, where stress arises from an imbalance between the demands placed on parents and the resources available to meet them (12, 50). The findings are consistent with previous research showing that child and parental characteristics and systemic conditions contribute to elevated parenting stress among parents of children with ASD (15, 17, 19). This is in line with the 2016 meta-synthesis by Ooi et al. (19) which identified four overarching themes in parents’ experiences-parenting, impact on the family, social impact, and interactions with health and educational services-all of which are reflected in our findings. However, whereas previous syntheses tend to address these domains separately, our findings highlight how these dimensions are interconnected and experienced as part of an overall caregiving burden in everyday life. Similarly, the review by Bonis et al. (15) and the meta-synthesis by Dharanidharan and Kuruveettissery (17) address aspects of parenting stress, particularly in relation to challenging behavior and access to support and services, while our study further emphasizes how these factors are embedded in and shape parents’ daily routines and lived experiences. Scandinavian studies to date have primarily focused on parents’ experiences with receiving their child’s diagnosis (32, 43, 46), with preschool settings (45), and with societal support and child health care services (44, 47). In contrast, the present study contributes by capturing parenting stress as part of a broader, everyday caregiving burden, as it unfolds across home life, family routines, social participation, and interactions with welfare services within a Scandinavian context.

Our study shows that several parental activities spanned multiple ecological layers of Bronfenbrenner (48, 49), with a key cross-level task being educating siblings, extended family, peers, preschool staff, and service providers about ASD and the child’s needs. Parents described limited knowledge and assumptions among others, leading to misunderstandings that required them to repeatedly guide and explain. The informational need identified by our parents aligns with Li et al. (20), who identified similar misunderstandings within extended families due to insufficient ASD knowledge. Our parents also reported limited competence in preschool and welfare services, consistent with Swedish studies documenting comparable information gaps in preschools (45), habilitation and relief services (44) and child healthcare (47). Aligned with prior research, our results highlight a substantial and cumulative informational burden across ecological layers, that contributes to parental stress (12, 13).

Parents in this study valued both practical and emotional support across ecological layers of Bronfenbrenner (48, 49). Family, friends, other parents, preschool staff, and various service providers were key sources of help. Extended family, especially grandparents and relatives familiar with ASD, provided essential everyday support. These findings are consistent with Li et al. (20) highlighting the role of extended family in the lives of persons with ASD. Parents also described individuals with relevant experience becoming important allies, offering guidance and a safe, understanding space. These findings align with Oii et al. (19) and Gustafsson and Sund-Levander (47), who emphasize the importance of sharing experiences with other parents or empathic professionals. The significance of social capital is supported by studies on parental stress (15), like Ooi et al. who highlighted value of practical and emotional support from other parents having similar experiences and being able to share problems that others might not understand (19), showing that social support serves as a protective factor for parental coping (16).

Across Bronfenbrenner`s ecological layers (48, 49), parents faced challenges participating in social activities at home, with family and friends, and in preschool settings. Several parents described hosting at home easier, and they frequently had to interpret the child’s signals, structure situations, use distraction or sensory materials, and adapt timing and sensory environments. These findings align with O’Nions et al. (28), who document similar demands on parental flexibility and environmental adaptation. Parents also reported feelings of isolation, consistent with previous research (15, 19). An interesting contribution from our study is parents’ preference for informal outdoor activities, which provide manageable and low-demand social opportunities. Another novel finding concerns the challenges parents experienced when attending preschool activities together with other parents, events that represent important social arenas in Scandinavian societies. Overall, these findings reveal the cumulative care burden and the multidimensional nature of parenting and parenting stress across ecological levels for families raising a young child with ASD.

### Parenting stress in Scandinavian family life

This study highlights underexplored aspects of parenting stress by Abidin (12, 50) that emerge from the dynamic interplay between child characteristics, parental factors, and broader social environments within everyday Scandinavian family life when raising a young child with ASD. Parents described a need for continuous adaptation to developmental and behavioral demands, resulting in extensive planning, rigid routines, and highly child−centered patterns, consistent with findings of O`Nions et al. (28) and Bonis et al (15). Emotional strain, developmental concerns, and the division of caregiving responsibilities to meet sibling needs also reflected patterns identified elsewhere (19). Consistent with our findings, Ooi et al.’s meta-synthesis (19) showed that parents often described dividing family resources to meet the needs of both siblings and the child with ASD, often accompanied by feelings of worry or guilt about reduced time and attention for siblings. While these dynamics are well established globally, they offer new insight into how caregiving unfolds within Scandinavian families. Parents experienced high demands in parenting, contributing to substantial parental stress (12, 50) and persistent worry. Yet, parents also reported gradually developing competence and resilience through trial and error. Overall, the findings correspond with international research highlighting the critical role of early guidance and family support (29, 32, 44), yet they also add context−specific knowledge from a Scandinavian welfare setting. Notably, even with formal entitlements to support, parents in our Scandinavian context report caregiving experiences that mirror those reported in broader international syntheses (17, 19). This may suggest that the availability of services does not necessarily translate into support that sufficiently addresses the demands of everyday family life; from the perspective of Abidin’s model (12, 50), parenting stress may persist when there is an imbalance between these demands and the resources experienced as available and meaningful in daily life.

Another key finding was parents’ safety−related stress, both at home and in public. Parents described persistent hypervigilance driven by fears related to water, traffic, heights, sharp objects, and the risk of elopement. Water−related concerns are well documented by Cosart et al. (52), who identify drowning as the leading cause of death in children with ASD and show how such fears shape family routines and emotional strain. O’Nions et al. (28) also reported safety challenges similar to those in our study, like continuous monitoring and environmental adaptations such as locking doors, installing alarms, or restricting access to dangerous items. As far as we know, no Scandinavian studies have addressed this concern in family life, but in the study by Andersson et al. parents reported concerns of safety in preschools (45). Our findings align with this international evidence while extending it: parents in our study additionally emphasized worries about heights during outdoor activities, such as hiking, an activity many Norwegians spend a lot of time doing. Another new finding was the strain of feeling to know what the child was doing, even in another room. Such pervasive vigilance based on the child characteristics, contributes to elevated parenting stress described by Abdin (12, 50), with potential impacts on mental and physical health. These findings underscore the parental burden and parental stress in family life and the need for greater awareness among both professionals and the public to secure support for these families.

### The impact of preschool and welfare services on parenting stress and family life

Parents in this study described highly variable preschool experiences with their children. Some experienced competent, stable staff who understood their child, offered adapted support, and provided guidance, which appeared to be crucial for reducing parental stress (12, 50) and fostering trust. These findings align with earlier research (19), including studies from Scandinavian preschool settings in Sweden (44, 45, 47). Andersson et al. (2021) described parental experiences from Swedish preschools, emphasizing the staff’s need for education and experience in working with children with ASD, as well as the importance of commitment and regular dialogue with parents (45). Similarly, Gustafsson et al. (2024) highlighted the significance of confidence and collaboration, alongside access to information and professional competence (47). Concerns about the child’s development and the amount of time spent in preschool while parents are working were also reflected in both our study and the Swedish study by Andersson et al. (45).

A new contribution from this study is the central importance parents attributed to the special needs teacher as a key source of expertise and stability, complementing Scandinavian findings on the vital role of preschool and societal support systems (44, 45, 47). In our study, well-functioning collaboration was linked to parental relief and heightened hope for the child’s development. This is consistent with Ooi et al. (19), who found that child improvement fostered hope among parents. Unlike our study, however, parents also reported adjusting their expectations and hopes, which may be related to their inclusion of parents of children up to 12 years of age, who are at a later stage in their child’s developmental trajectory.

Another finding in our study was that when preschool services were experienced as inadequate, due to poor adaptation, instability, or lack of competence, parents reported significant spillover effects at home, including disrupted eating and drinking, increased distress, and a need for parents to leave work. Parents also highlighted mornings as particularly vulnerable periods requiring predictability, time, and flexibility to secure successful transitions. Overall, the study shows that a social system factor like preschool functioning is a major determinant of parenting stress as described by Abidin (12, 50), with high-quality services playing a critical role in supporting parental resilience and family well-being (53, 54).

Parents in our study also described fragmented and inconsistent support across systems, which substantially increased parenting stress as described by Abidin (12, 50). Some families reported improvements only after relocating or changing preschools, reflecting Scandinavian findings from both Swedish and Danish research on geographic, systemic, and competence-related variation in service quality (32, 43, 44, 47). Both in Andersson et al. (2017) and in our study parents describe better services after moving to another municipality. Bureaucratic barriers, complex applications, and the constant need to “chase” or “fight” for services left many feeling exhausted and overwhelmed, paralleling findings by Andersson et al. (2017) on fighting hard for the child’s rights as well as chasing support (44). In our study, some parents even used war metaphors and questioned the reliability of the welfare state when support was insufficient or poorly coordinated, experiences that intensified stress and reduced trust in services. Similar concerns regarding lack of trust in preschool services have been reported by parents in studies by Andersson et al. (2021) (45) and Gustafsson et al. (2024) (47). In addition, Carlsson et al. (2016) (32) highlighted the challenges parents face in trusting professionals during the support process. Trust in caregivers and support providers appears to be essential for parents, as lack of trust contributes to increased worry and parental stress. Lack of flexibility and individual adaptation led some parents in our study to decline relief services they viewed as unsuitable, echoing dissatisfaction with low-competence relief services among Swedish parents (44). The lack of individualization aligns with experiences in the same Swedish study by Andersson et al. (2017), but the need for flexibility is not addressed in the same way. Parents in our study consistently called for ASD-specific expertise and individualized support tailored to the family’s needs, including in emergencies.

Our finding of the need for early assignment of a competent coordinator immediately after diagnosis aligns with findings on poor coordination in Scandinavian systems (44, 47). Parents in our study described feeling particularly alone in the period immediately following the diagnosis, when they were struggling to process a heavy message while simultaneously having to navigate a complex welfare system.

This experience mirrors patterns reported in Swedish research, where parents similarly described a lack of support and guidance during early stages of service involvement (44). Several parents also emphasized the bureaucratic challenges involved in applying for a coordinator, including delays in allocation and the need to appeal decisions, further exacerbating feelings of vulnerability and isolation. These findings underscore how systemic shortcomings directly amplify parenting stress as described by Abidin (12, 50).

### Strengths and limitations

This study has several strengths that should be highlighted. It offers an in-depth exploration of parenting among parents of young children (3–5 years old) with ASD in Norway and draws on a broad range of parental experiences. The sample included diversity in parental educational background and time since the child’s diagnosis, as well as variation in the child’s functional level. Both mothers and fathers were included, and interviews were conducted prior to participation in a parenting program, which allowed for the collection of rich and nuanced data not influenced by intervention experience. 

However, the study also has limitations. The sample included only parents who were able and willing to participate in the ENACT study and who were proficient in the Norwegian language. Multilingual families participated, albeit in limited numbers. Recruitment from an ongoing intervention study may have introduced selection bias, as participating parents are likely to be particularly engaged with services. This may limit the transferability of the findings to parents with less contact or engagement with support services. As expected, given the gender distribution of the diagnosis, few girls were represented in the sample, despite targeted efforts to increase their participation during recruitment. A limitation of systematic text condensation is its reliance on the researcher’s interpretation throughout the analytic process, which may introduce subjectivity and influence how meaning is constructed from the data; to mitigate this, we worked collaboratively as a team and maintained transparency regarding our individual preunderstandings.

The findings should be understood within the Scandinavian welfare context, characterized by universal service provision and decentralized municipal responsibility. While this model provides broad access to services, it may also contribute to fragmentation, variation in service quality, and considerable administrative demands on parents, shaping experiences of coordination and stress following an ASD diagnosis. Accordingly, the findings may not be fully transferable to market-based or insurance-driven systems. However, challenges related to coordination, system navigation, and the need for early, family-centred support have been reported across diverse contexts, suggesting that key aspects of parental burden may extend beyond the Scandinavian welfare setting.

### Implications

This study highlights the need for holistic, flexible, and equitable services systems for families of children with ASD. Timely and competent post-diagnostic support, including clear guidance, is essential for establishing stable family routines and preventing escalating parental stress. In line with this, safety challenges should be proactively identified through home assessments and environmental adaptations conducted by ASD-competent staff. The findings also emphasize the importance of tailored support for siblings and extended family, as well as competence-building initiatives within the broader social network to reduce parental burden. High-quality preschool services are a key component of early support, playing a vital role in promoting child development, supporting parents and reducing parental stress.

Professionals across sectors must have access to ASD-spesific training and ongoing competence development to ensure consistent and appropriate service provision. Municipal services should be stable, adaptable, and well-coordinated, actively incorporating parental expertise and ensuring access to respite care as well as practical and emotional support. Designated and competent child coordinators play an essential role in facilitating coherent, individualized, and continuous service pathways across sectors. Early coordination and support should be mandated as a standard post-diagnostic service, rather than depending on parental advocacy or municipal discretion. Investing in early and coordinated services may prevent long-term costs, by reducing parental stress, service breakdown, and later, more intensive interventions.

Finally, the study underscores the need for clear national guidelines and standards, as the rising prevalence of ASD places increasing demands on local welfare infrastructures and underscores the importance of ensuring equitable service provision regardless of municipality.

## Conclusion

Parenting a child with ASD entails ongoing emotional regulation, adaptation, and learning that permeate daily family life. Parental adaptation is shaped by both the child’s needs and the coherence, or fragmentation, of surrounding service systems. Insufficient competence, flexibility, or coordination in services increases parental burden, whereas effective support, moments of mastery, and collaborative partnerships foster resilience and positive family trajectories. Understanding how these dynamics unfold across home and service contexts is crucial for developing interventions that are competent, adaptable, and aligned with family realities. Although the Scandinavian welfare model provides extensive support, gaps in practice may intensify rather than alleviate stress. More coherent and responsive service structures are needed to ensure timely, appropriate, and family-sensitive support. Future research should examine how service design, collaboration, and early intervention can better reflect parents’ lived experiences and promote long-term family well-being.

## Data Availability

The original contributions presented in the study are included in the article/**Supplementary Material**, further inquiries can be directed to the corresponding author/s.

## References

[1] American Psychiatric Association . Diagnostic and statistical manual of mental disorders. Arlington, Va, USA: American Psychiatric Association (2013).

[2] Desquenne Godfrey G Downes N Cappe E . A systematic review of family functioning in families of children on the autism spectrum. J Autism Dev Disord. (2024) 54:1036–57. doi: 10.1007/s10803-022-05830-6 36626001

[3] Zeidan J Fombonne E Scorah J Ibrahim A Durkin MS Saxena S . Global prevalence of autism: A systematic review update. Autism Res. (2022) 15:778–90. doi: 10.1002/aur.2696 35238171 PMC9310578

[4] Sacco R Camilleri N Eberhardt J Umla-Runge K Newbury-Birch D . The prevalence of autism spectrum disorder in Europe. In: Carotenuto M , editor.Autism spectrum disorders-recent advances and new perspectives. London, UK: IntechOpen (2023). p. 1–16.

[5] Posserud MB Skretting Solberg B Engeland A Haavik J Klungsøyr K . Male to female ratios in autism spectrum disorders by age, intellectual disability and attention‐deficit/hyperactivity disorder. Acta Psychiatr Scand. (2021) 144:635–46. doi: 10.1111/acps.13368 34494265

[6] Narzisi A Vivanti G Berloffa S Milone A Fantozzi P Tancredi R . Sensory processing in autism: A call for research and action. Front Psychiatry. (2025) 16:1584893. doi: 10.3389/fpsyt.2025.1584893 40469378 PMC12135051

[7] Tecar C Chiperi LE Iftimie BE Popa LL Sas V Stefanescu E . Sleep disturbances and behavioral problems in children and adolescents with autism spectrum disorder - a systematic review. Clinics Pract. (2025) 15:201. doi: 10.3390/clinpract15110201 41294632 PMC12651674

[8] Riccio MP Marino M Garotti R Tassiello A Maffettone V Pezone M . Food selectivity in autism spectrum disorder: Implications of eating, sensory and behavioural profile. Front Psychiatry. (2025) 16:1587454. doi: 10.3389/fpsyt.2025.1587454 40530068 PMC12171170

[9] Baraskewich J von Ranson KM McCrimmon A McMorris CA . Feeding and eating problems in children and adolescents with autism: A scoping review. Autism. (2021) 25:1505–19. doi: 10.1177/13623613211995631 PMC8323334 33653157

[10] McConkey R Truesdale‐Kennedy M Cassidy A . Mothers’ recollections of early features of autism spectrum disorders. Child Adolesc Ment Health. (2009) 14:31–6. doi: 10.1111/j.1475-3588.2008.00495.x 40046247

[11] Adams D Dargue N Paynter J . Longitudinal studies of challenging behaviours in autistic children and adults: A systematic review and meta-analysis. Clin Psychol Rev. (2023) 104:1–19. doi: 10.1016/j.cpr.2023.102320 37515997

[12] Abidin RR . The determinants of parenting behavior. J Clin Child Psychol. (1992) 21:407–12. doi: 10.1207/s15374424jccp2104_12 42071266

[13] Deater-Deckard K . Parenting stress and child adjustment: Some old hypotheses and new questions. Clin Psychol: Sci Pract. (1998) 5:314–32. doi: 10.1111/j.1468-2850.1998.tb00152.x 40046247

[14] Hayes SA Watson SL . The impact of parenting stress: A meta-analysis of studies comparing the experience of parenting stress in parents of children with and without autism spectrum disorder. J Autism Dev Disord. (2013) 43:629–42. doi: 10.1007/s10803-012-1604-y 22790429

[15] Bonis S . Stress and parents of children with autism: A review of literature. Issues Ment Health Nurs. (2016) 37:153–63. doi: 10.3109/01612840.2015.1116030 27028741

[16] Turnage D Conner N . Quality of life of parents of children with autism spectrum disorder: An integrative literature review. J Spec Pediatr Nurs. (2022) 27:Article 12391. doi: 10.1111/jspn.12391 35986656

[17] Dharanidharan D Kuruveettissery S . Parental perspectives on stress and challenges in raising autistic children: A meta-synthesis. J Psychosoc Rehabil Ment Health. (2024) 12:125–41. doi: 10.1007/s40737-024-00420-4 30311153

[18] Oliver M Poysden Z Gillespie-Smith K . A qualitative systematic review and meta-synthesis of mothers’ experiences of parenting autistic women and girls. Rev J Autism Dev Disord. (2024), 1–24. doi: 10.1007/s40489-024-00472-z 30311153

[19] Ooi Ong YS Jacob SA Khan TM . A meta-synthesis on parenting a child with autism. Neuropsychiatr Dis Treat. (2016) 12:745–62. doi: 10.2147/NDT.S100634 27103804 PMC4827600

[20] Li J-L Washington-Nortey M Kifle TH Cotier F Hoekstra RA . The role of extended family members in the lives of autistic individuals and their parents: A systematic review and meta-synthesis. Clin Child Family Psychol Rev. (2025) 28(2):507–39. doi: 10.1007/s10567-025-00525-7 40392445 PMC12162707

[21] Cervellione B Iacolino C Bottari A Vona C Leuzzi M Presti G . Functioning of neurotypical siblings of individuals with autism spectrum disorder: A systematic review. Psychiatry Int. (2025) 6:52. doi: 10.3390/psychiatryint6020052 30654563

[22] Karst JS Van Hecke AV . Parent and family impact of autism spectrum disorders: A review and proposed model for intervention evaluation. Clin Child Family Psychol Rev. (2012) 15:247–77. doi: 10.1007/s10567-012-0119-6 22869324

[23] Gholipour K Ghiasi A Shahrokhi H Dadashi Z Javanmard S Tabatabaei SH . Perceptions of the professionals and parents of children with autism spectrum disorders about autism services; a qualitative study. J Autism Dev Disord. (2023) 53:96–109. doi: 10.1007/s10803-021-05388-9 34982323

[24] Mo S Li S Jin Z . Parental stress and coping strategies in parents of children with autism spectrum disorders: A systematic review and meta-analysis. Rev J Autism Dev Disord. (2025) 1–21. doi: 10.1007/s40489-025-00509-x 30311153

[25] Nkgaphela MR Sumbane GO Hlahla LS Mokhwelepa LW . Parenting on the margins: A qualitative study of the challenges encountered by fathers of children with autism in Limpopo Province, South Africa. Front Psychiatry. (2025) 16:1730241. doi: 10.3389/fpsyt.2025.1730241 41561983 PMC12812744

[26] Legg H Tickle A . Uk parents’ experiences of their child receiving a diagnosis of autism spectrum disorder: A systematic review of the qualitative evidence. Autism. (2019) 23:1897–910. doi: 10.1177/1362361319841488 30995082

[27] Salleh NS Abdullah KL Yoong TL Jayanath S Husain M . Parents’ experiences of affiliate stigma when caring for a child with autism spectrum disorder (Asd): A meta-synthesis of qualitative studies. J Pediatr Nurs. (2020) 55:174–83. doi: 10.1016/j.pedn.2020.09.002 32957021

[28] O’Nions E Happé F Evers K Boonen H Noens I . How do parents manage irritability, challenging behaviour, non-compliance and anxiety in children with autism spectrum disorders? A meta-synthesis. J Autism Dev Disord. (2018) 48:1272–86. doi: 10.1007/s10803-017-3361-4 29222612 PMC5861158

[29] Boshoff K Gibbs D Phillips RL Wiles L Porter L . A meta‐synthesis of how parents of children with autism describe their experience of advocating for their children during the process of diagnosis. Health Soc Care Community. (2019) 27:143–57. doi: 10.1111/hsc.12691 30548710

[30] Espinosa-Díaz N Vega-Arce M Jara MI Garrido F . Stress in Parents of Preschoolers with Autism Spectrum Disorders: An Update Review. Bol Med Hosp Infant Mex. (2024) 81(4):195–209. doi: 10.24875/BMHIM.23000182 39236670

[31] Huang M Zhou Z . Factors contributing to parental stress among Chinese families of children with autism: A qualitative study. Psychol Sch. (2023) 60:1837–54. doi: 10.1002/pits.22837 41531421

[32] Carlsson E Miniscalco C Kadesjö B Laakso K . Negotiating knowledge: Parents’ experience of the neuropsychiatric diagnostic process for children with autism. Int J Lang Commun Disord. (2016) 51:328–38. doi: 10.1111/1460-6984.12210 26833425

[33] Rostgaard T . Family policies in scandinavia. Berlin: Fredrich-Ebert-Stiftung (2014). Available online at: https://library.fes.de/pdf-files/id/11106.pdf (Accessed February 16, 2026).

[34] Statistics Norway . Labour force survey: statistics Norway (2026). Available online at: https://www.ssb.no/en/arbeid-og-lonn/sysselsetting/statistikk/arbeidskraftundersokelsen (Accessed February 11, 2026).

[35] Statistics Norway . How many fathers take paternity leave (2026). Available online at: https://www.ssb.no/en/befolkning/likestilling/statistikk/indikatorer-for-kjonnslikestilling-i-kommunene/Articles%20for%20Indicators%20for%20gender%20equality%20in%20municipalities/how-many-fathers-take-paternity-leave (Accessed February 13, 2026).

[36] Ministry of Education and Research . Early Childhood Education and Care (2023) [cited 2026 April 13]. Available online at: https://www.regjeringen.no/en/topics/families-and-children/kindergarden/early-childhood-education-and-care-polic/id491283/

[37] Statistics Norway . Statistics on kindergartens: statistics Norway (2025). Available online at: https://www.ssb.no/en/utdanning/barnehager/statistikk/barnehager (Accessed February 13, 2026).

[38] The Norwegian Directorate for Education and Training . Special education support – facts about preschools 2024 (2024). Available online at: https://www.udir.no/tall-og-forskning/statistikk/statistikk-barnehage/analyser/2025/fakta-om-barnehager-2024/spesialpedagogisk-hjelp/ (Accessed February 13, 2026).

[39] Skorpen S Søndenaa E . Norwegian perspectives on health care for people with intellectual and developmental disabilities. J Policy Pract Intellect Disabil. (2024) 21:e12492. doi: 10.1111/jppi.12492 40046247

[40] Povlsen L Karlsson LE Regber S Sandstig G Fosse E . Are equity aspects communicated in Nordic public health documents? Scand J Public Health. (2014) 42:235–41. doi: 10.1177/1403494813520358 24492675

[41] Lovdata . Lov om kommunale helse- og omsorgstjenester (2011). Available online at: https://lovdata.no/dokument/NL/lov/2011-06-24-30/KAPITTEL_3#KAPITTEL_3 (Accessed February 13, 2026).

[42] Nordic Health & Welfare Statistics . Services to people with disabilities: nordic health & Welfare statistics (2025). Available online at: https://nhwstat.org/welfare/disability/services-people-disabilities (Accessed February 16, 2026).

[43] Rasmussen PS Pedersen IK Pagsberg AK . Biographical disruption or cohesion?: How parents deal with their child’s autism diagnosis. Soc Sci Med. (2020) 244. doi: 10.1016/j.socscimed.2019.112673 31735475

[44] Andersson GW Miniscalco C Gillberg N . A 6-year follow-up of children assessed for suspected autism spectrum disorder: Parents’ experiences of society’s support. Neuropsychiatr Dis Treat. (2017) 13:1783–96. doi: 10.2147/ndt.S134165 28744128 PMC5511026

[45] Andersson GW Gillberg N Miniscalco C . Parents of children diagnosed with autism spectrum disorder: What do they expect and experience from preschools? Neuropsychiatr Dis Treat. (2021) 17:3025–37. doi: 10.2147/ndt.S324291 34611403 PMC8487270

[46] Andersson GW Miniscalco C Gillberg C . Preschoolers assessed for autism: Parent and teacher experiences of the diagnostic process. Res Dev Disabil. (2014) 35:3392–402. doi: 10.1016/j.ridd.2014.08.027 25194515

[47] Gustafsson BM Sund-Levander M . Parents’ experiences of investigations and interventions by child healthcare, child and adolescent psychiatry and child and youth habilitation. Child Health Care. (2024) 53:1–22. doi: 10.1080/02739615.2022.2143360 37339054

[48] Bronfenbrenner U . The ecology of human development: experiments by nature and design. Cambridge, MA, USA: Harvard University Press (1979).

[49] Bronfenbrenner U Morris PA . The bioecological model of human development. In: Lerner RM Damon M , editors.Handbook of child psychology: theoretical models of human development. Hoboken, NJ, USA: John Wiley & Sons, Inc (2007). p. 793–828.

[50] Abidin RR . Parenting stress index. Professional manual. Odessa, FL: Psychological Assessment Resources (1995).

[51] Malterud K . Systematic text condensation: A strategy for qualitative analysis. Scand J Public Health. (2012) 40:795–805. doi: 10.1177/1403494812465030 23221918

[52] Cosart BD Lawson KA Williams SR Lewis KE Namutebi R Johnson MB . Parent perspectives on water safety for children with autism. J Autism Dev Disord. (2025), 1–10. doi: 10.1007/s10803-025-06819-7 40237850 PMC13427885

[53] Ilias K Cornish K Kummar AS Park M-A Golden KJ . Parenting stress and resilience in parents of children with autism spectrum disorder (Asd) in Southeast Asia: A systematic review. Front Psychol. (2018) 9:Article 280. doi: 10.3389/fpsyg.2018.00280 29686632 PMC5900388

[54] Rusu PP Candel O-S Bogdan I Ilciuc C Ursu A Podina IR . Parental stress and well-being: A meta-analysis. Clin Child Family Psychol Rev. (2025) 28:255–74. doi: 10.1007/s10567-025-00515-9 40057656 PMC12162691

