# Supplementary material for: The burden of care, parenting stress, and navigating welfare services: parents’ everyday experiences of young children with autism spectrum disorder
Source: Front Psychiatry. 2026 Jun 30;17:1841274. doi: 10.3389/fpsyt.2026.1841274 (PMC13366122; doi:10.3389/fpsyt.2026.1841274)
Supplement: Supplementary file 1 [file Table1.docx]

**Supplementary 1** Interview guide

| Questions |
| --- |
| How do you experience being a parent of a child with autism?  Are there specific challenges related to being a parent of a child with autism?  What do you find necessary for having a good day together with your child?  When do you have the best moments with your child?  What can make things difficult for you and your child?  How do you experience your child’s difficulties with language and social interaction?  How do you experience the connection with your child? What kind of feedback do you receive?  Does your child struggle with adapting? How does this affect you as a parent?  What makes you happy? What makes you worried?  How are you and your child met by people around you?  Who do you turn to for help and support?  How does having a child with autism affect family life in general (siblings and extended family)?  How does having a child with autism affect other areas of your life, such as work, education, leisure, and participation in activities?  What do you need as a parent to have a good everyday life? |
